# Pd/C-Mediated synthesis of indoles in water

**DOI:** 10.3762/bjoc.5.46

**Published:** 2009-09-23

**Authors:** Mohosin Layek, Udaya Lakshmi, Dipak Kalita, Deepak Kumar Barange, Aminul Islam, K Mukkanti, Manojit Pal

**Affiliations:** 1 Dr. Reddy’s Laboratories Ltd, Bollaram Road, Miyapur, Hyderabad 500049, India; 2 Chemistry Division, Institute of Science and Technology, JNT University, Kukatpally, Hyderabad 500072, India; 3 New Drug Discovery, R&D Center, Matrix Laboratories Ltd., Anrich Industrial Estate, Bollaram, Jinnaram Mandal, Medak District, Andra Pradesh 502 325, India; 4 (present address:) Institute of Life Science, University of Hyderabad Campus, Gachibowli, Hyderabad 500 046, Andhra Pradesh, India

**Keywords:** C–C bond, charcoal, copper, indoles, palladium

## Abstract

We describe the utility of a Pd/C-Cu mediated method in the synthesis of 2,5-disubstituted indoles in water via a coupling-cyclization strategy. Further application of this methodology has been demonstrated in the preparation of a target indole derivative via a 7-step process the key step being the Pd/C-mediated coupling reaction.

## Introduction

**Figure 1:**
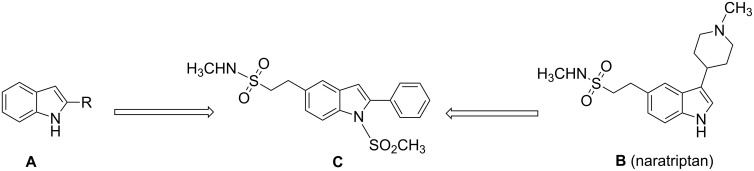
Design of novel indole derivative **C** of pharmacological interest.

2-Substituted indoles (**A**, Figure 1) display a wide range of pharmacological activities and therefore have been explored as a number of potential therapeutic agents [1] e.g. inhibitors of proteases involved in coagulation [2–3], antagonists of G-protein-coupled receptors [4–5], anti-angiogenic compounds [6] and inhibitors of endothelinconverting-enzyme [7]. 5-Alkyl substituted indoles e.g. naratriptan (**B**, Figure 1) on the other hand have been reported as a 5-HT_1B/1D_ receptor agonists for the potential treatment of migraine headache [8]. Thus a combination of both in a single molecule was expected to provide novel agent (**C**, Figure 1) of potential biological significance. Due to our continued interest in the development of indole based compound libraries we decided to synthesize compound **C** and its analogues.

A large number of methods [9–12] have been reported for the construction of indole ring many of which are mediated by transition metal catalysts. Among these methods palladium mediated protocols have gain considerable interest [13]. In our effort towards the development of palladium-catalyzed reactions in aqueous media, we have reported an efficient synthesis of 2-substituted indoles via a Pd/C-catalyzed coupling-cyclization process in water [14]. Herein, we report a further continuation of our previous work towards the synthesis of a variety of indole derivatives along with the preparation of target compound **C**.

## Results and Discussion

**Scheme 1:**
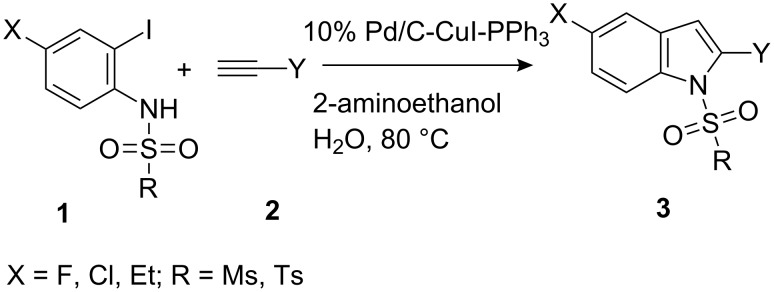
Preparation of 2,5-disubstituted indole.

In our previous study after examining a number of *N*-substituted *o*-iodoanilides we have observed that the *N*-methanesulfonyl derivative provided the best result in terms of product nature and yields [14]. However, the presence of only one substituent, i.e. a methyl group on the *o*-iodoaniline ring was examined. Moreover, the presence of other sulfonyl groups e.g. the benzenesulfonyl moiety on the aniline nitrogen was not studied. In the present study we aimed to conduct further research taking into account of above-mentioned aspects. Thus, when *o*-iodoanilide **1** was treated with terminal alkyne **2** (3.0 equiv) in water in the presence of 10% Pd/C (0.03 equiv), PPh_3_ (0.12 equiv), CuI (0.06 equiv) and 2-aminoethanol (3.0 equiv) under nitrogen, 2-substituted indoles **3** were obtained in good yields (Scheme 1). The details of this study are summarized in Table 1.

**Table 1:** Pd/C-Catalyzed synthesis of 2,5-disubstituted *N*-sulfonyl-indoles in aqueous media.^a^

Entry	*o*-Iodoanilide **1**	Alkyne **2**; Y =	Time (h)	Product **3**^b^	Yield (%)^c^

1.	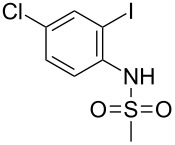 **1a**	-CH_2_CH(OH)CH_3_ **2a**	10	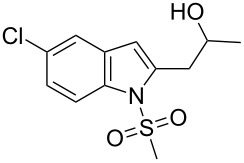 **3a**	79
2.	**1a**	-C_6_H_4_CH_3_-*p* **2b**	4	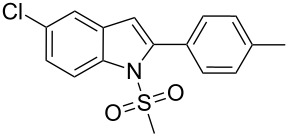 **3b**	86
3.	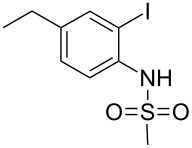 **1b**	**2b**	6	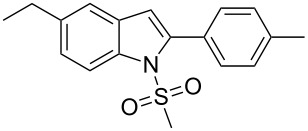 **3c**	90
4.	**1b**	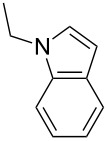 **2c**	10	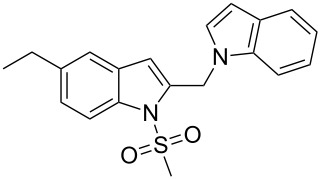 **3d**	75
5.	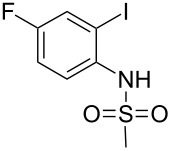 **1c**	**2b**	12	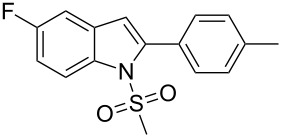 **3e**	78
6.	**1c**	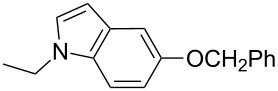 **2d**	14	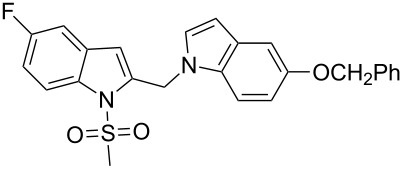 **3f**	90
7.	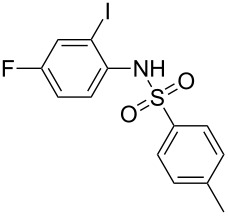 **1d**	**2c**	10	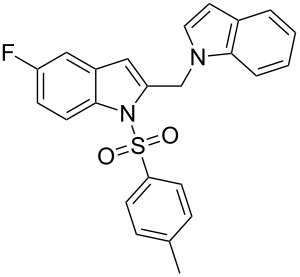 **3g**	76
8.	**1d**	-(CH_2_)_3_OH **2e**	14	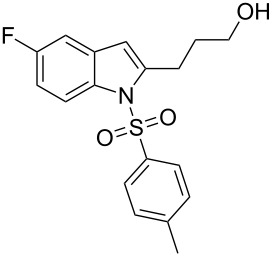 **3h**	80
9.	**1d**	-CH_2_CH(OH)CH_3_ **2a**	12	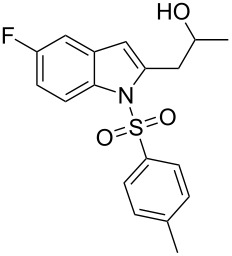 **3i**	65
10.	**1d**	-C(CH_3_)_2_OH **2f**	10	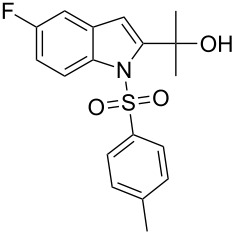 **3j**	70
11.	**1c**	**2c**	14	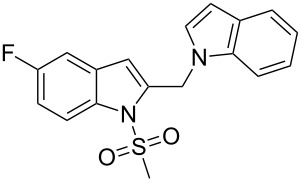 **3k**	75
12.	**1d**	**2b**	10	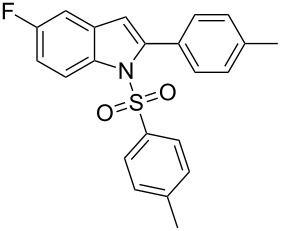 **3l**	78
13.	**1d**	-C_6_H_5_ **2g**	8	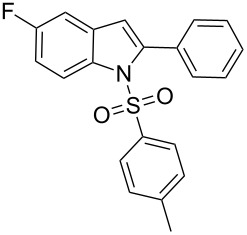 **3m**	68
14.	**1d**	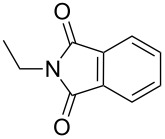 **2h**	12	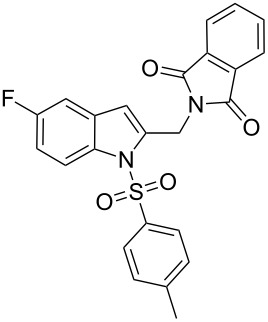 **3n**	70
15.	**1d**	-(CH_2_)_3_CH_3_ **2i**	12	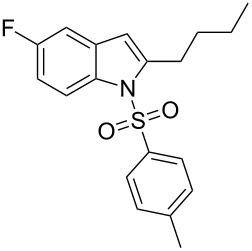 **3o**	74
16.	**1c**	-C_6_H_4_NO_2_-*o* **2j**	24	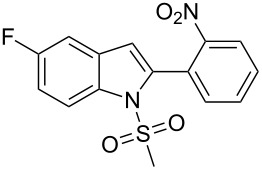 **3p**	78
17.	**1c**	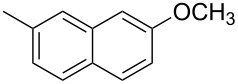 **2k**	12	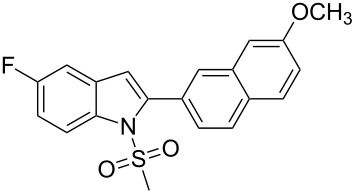 **3q**	78
18.	**1c**	-(CH_2_)_3_Cl**2l**	5	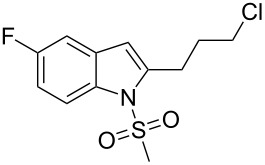 **3r**	74
19.	**1c**	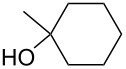 **2m**	3	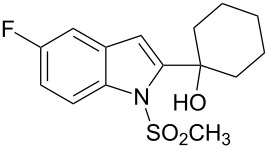 **3s**	85

^a^All reactions were carried out by using **1** (1.0 equiv), **2** (3.0 equiv), 1:4:2 ratio of Pd/C:PPh_3_:CuI, 2-aminoethanol (3.0 equiv) in H_2_O. ^b^Identified by ^1^H NMR, ^13^C NMR, IR, MS. ^c^Isolated yields.

We have examined four anilides **1a–d** each of which participated well in the present Pd/C-mediated coupling-cyclization process. Substituents such as halogen atoms (**1a**, **1c** and **1d**) and alkyl groups (**1b**) on the aryl ring were well tolerated. A range of terminal alkynes **2** were employed in the present reaction to obtain the corresponding indole **3** in 65–90% yield via a single step method. Thus, we have extended the generality and scope of our previously reported method considerably demonstrating its potential in the synthesis of a diversity based indole library.

In our previous [14] as well as the present study we have used three equivalents of terminal alkynes to obtain the optimum yields of products. It is possible that the excess equivalents of terminal alkynes might undergo dimerization via oxidative coupling though no significant amount of 1,3-butadiyne derivative was isolated as a side product from any of the reactions presented in Table 1. However, to assess the effect of using fewer equivalents of terminal alkynes we conducted an experiment using **1a** and **2a** in a ratio of 1.0:1.2 without changing the other reaction conditions. The desired indole **3a** was isolated in 60% yield (vs 79% of entry 1, Table 1) confirming the requirement for an excess amount of terminal alkyne to achieve the best yield of the desired product.

We have also examined the use of a basic iodoaniline such as *N*-methyl substituted 2-iodoaniline in the present coupling reaction with terminal alkynes. Isolation of 2-alkynyl substituted *N*-methylaniline as a sole product rather than expected indole indicated that the presence of an electron donating group on the nitrogen was unfavourable for the cyclization step though the C–C coupling reaction proceeded well under the conditions employed.

**Scheme 2:**
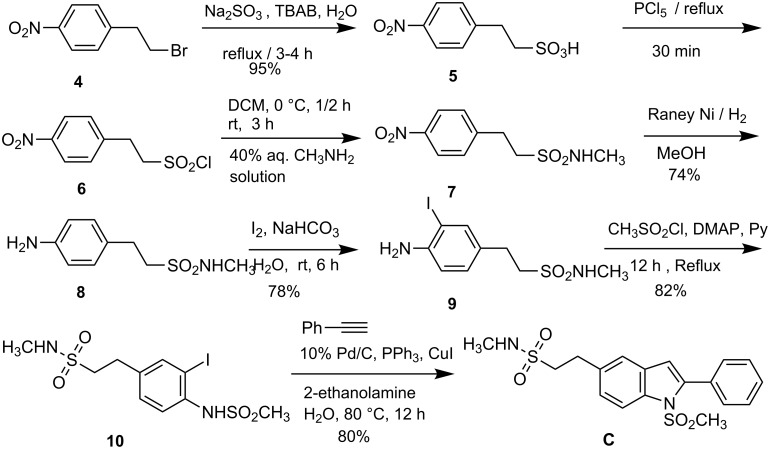
Preparation of compound **C**.

Having prepared a variety of *N*-substituted indole derivatives successfully we then undertook the synthesis of the target indole **C**. Compound **C** was prepared from bromo compound **4** following a 7-step process using the Pd/C-mediated indole synthesis as a key step as shown in Scheme 2. Thus treatment of bromide **4** with Na_2_SO_3_ provided corresponding sulfonic acid **5** which on treating with PCl_5_ provided the sulfonyl chloride derivative **6**. On reaction with aqueous methylamine compound **6** provided the methanesulfonamide derivative **7**, the nitro group of which was reduced in the presence of Raney nickel to yield the corresponding aniline derivative **8**. Iodination of **8** using elemental iodine provided the *o*-iodoaniline derivative **9** which was converted to the corresponding methanesulfonamide **10**. Compound **10** when reacted with phenylacetylene in the presence of 10% Pd/C-CuI-PPh_3_ and 2-aminoethanol in water provided the desired indole derivative **C**.

## Conclusions

In conclusion, we have demonstrated the potential of our previously reported palladium on carbon mediated practical synthesis of 2-substituted indoles in water. Besides preparing a wide variety of indole derivatives the methodology was utilized to prepare one of our target indole derivatives smoothly. We believe that the present demonstration would encourage organic/medicinal chemists to explore the utility of this methodology for easy access to indole based agents of pharmaceutical interest.

## Experimental

General methods: Unless stated otherwise, reactions were monitored by thin layer chromatography (TLC) on silica gel plates (60 F_254_), visualizing with ultraviolet light or iodine spray. Flash chromatography was performed on silica gel (60–120 mesh) using distilled petroleum ether and ethyl acetate. ^1^H and ^13^C NMR spectra were determined either in CDCl_3_ or DMSO-*d*_6_ solution using 400 and 50 MHz spectrometers, respectively. Proton chemical shifts (δ) are relative to tetramethylsilane (TMS, δ = 0.0) as internal standard and expressed in parts per million. Spin multiplicities are given as s (singlet), d (doublet), t (triplet), and m (multiplet) as well as b (broad). Coupling constants (*J*) are given in hertz. Infrared spectra were recorded on a FTIR spectrometer. Melting points were determined by using thermal analysis and differential scanning calorimetry (DSC) was generated with the help of DSC-60A dector. MS spectra were obtained on a mass spectrometer. Chromatographic (HPLC) purity was determined by using area normalization method and the condition specified in each case: column, mobile phase (range used), flow rate, detection wavelength, and retention times. All the reagents used are commercially available.

### General method for the preparation of *o*-iodoanilides (**1a–d**)

Step A (a typical procedure): A suspension of 4-fluoro aniline (5.0 g, 0.045 mol) in aqueous solution (50.0 mL) of sodium bicarbonate (0.045 mol) was stirred for 0.5 h. Then iodine (0.040 mol) was added at 5–10 °C and the mixture was stirred for 12.0 h. After completion, the reaction mixture was diluted with ethyl acetate (250 mL) and extracted with ethyl acetate (3 × 250 mL). The organic layers were collected, combined, washed with saturated aqueous sodium thiosulfate solution (2 × 125 mL), dried over anhydrous Na_2_SO_4_ and concentrated under vacuum. The crude product was purified by column chromatography on silica gel using 3:1, hexane\ethyl acetate to afford the desired product (6.4 g, 60% yield). 4-chloro-2-iodoaniline [15]: white solid, mp 42–43 °C; ^l^H NMR (CDCl_3_, 400 MHz) δ 7.59 (d, *J* = 2.2 Hz, 1H), 7.08 (dd, *J* = 8.6, 2.2 Hz, 1H), 6.62 (d, *J* = 8.6 Hz, 1H), 4.19 (br s, NH_2_); m/z (ES Mass) 253 (M+1, 100%); IR (cm^−1^, KBr) 3370, 1475, 1215, 1140. 4-ethyl-2-iodoaniline [16]: white solid, mp 38–41 °C; ^l^H NMR (CDCl_3_, 400 MHz) δ 7.45 (m, 1H), 6.95 (m, 1H), 6.66 (d, *J* = 8.0 Hz, 1H), 3.85 (br s, NH_2_), 2.58 (m, 2H), 1.25 (s, 3H); m/z (ES Mass) 247 (M+1, 100%); IR (cm^−1^, KBr) 3347, 1480, 1333, 1130. 4-fluoro-2-iodoaniline [17]: Orange oil; ^l^H NMR (CDCl_3_, 400 MHz) δ 7.35 (dd, *J* = 7.9, 2.2 Hz, 1H), 6.90–6.85 (m, 1H), 6.68–6.65 (m, 1H), 3.92 (br s, NH_2_); m/z (ES Mass) 237 (M+1, 100%); 3354, 1471, 1213, 1135.

Step B (a typical procedure): A mixture of 4-fluoro-2-iodo aniline (6.4 g, 0.027 mol), DMAP (0.49 g, 0.004 mol) was dissolved in pyridine (50.0 mL) and cooled to 0–5 °C. To this was added methanesulfonyl chloride (4.0 g, 0.035 mol) dropwise and the mixture was refluxed for 12 h. After completion of the reaction, the mixture was cooled to 5–10 °C, neutralized with dil HCl and extracted with ethyl acetate (3 × 300 mL). The organic layers were collected, combined, washed with saturated aq. NaCl (2 × 250 mL), dried over anhydrous Na_2_SO_4_ and concentrated under vacuum. The crude compound was purified by column chromatography on silica gel using 4:1 hexane\ethyl acetate to afford the desired product (5.1 g, 60% yield) as a brown solid,

*N*-(4-chloro-2-iodophenyl)methanesulfonamide [18] (**1a**): White solid, mp 111 °C; ^l^H NMR (CDCl_3_, 400 MHz) δ 7.76–7.85 (m, 2H), 7.06–7.11 (m, 1H), 6.51 (br s, 1H), 3.15 (s, 3H); m/z (ES Mass) 331 (M+1, 100%); 3255, 1480, 1335, 1165.

*N*-(4-ethyl-2-iodophenyl)methanesulfonamide (**1b**): White solid, mp 103 °C; ^l^H NMR (CDCl_3_, 400 MHz) δ 7.65 (d, *J* = 2.0 Hz, 1H), 7.53 (d, *J* = 8.2 Hz, 1H), 7.19 (dd, *J* = 8.0 and 2.0 Hz, 1H), 6.50 (br s, 1H), 2.98 (s, 3H) 2.57 (q, *J* = 7.6, 2H), 1.20 (t, *J* = 7.6, 3H); m/z (ES Mass) 325 (M+1, 100%); IR (cm^−1^, KBr) 3248, 1486, 1323, 1148.

*N*-(4-fluoro-2-iodophenyl)methanesulfonamide (**1c**): Brown solid, mp 91 °C; ^l^H NMR (CDCl_3_, 400 MHz) δ 7.55–7.65 (m, 2H), 7.10–7.15 (m, 1H), 6.47 (br s, 1H), 2.99 (s, 3H); m/z (ES Mass) 315 (M+1, 100%); 3254, 1481, 1330, 1155.

*N*-(4-fluoro-2-iodophenyl)benzenesulfonamide [17] (**1d**): Brown solid, mp 99 °C; ^l^H NMR (CDCl_3_, 400 MHz) δ 7.57–7.66 (m, 3H), 7.35–7.38 (m, 1H), 7.21–7.26 (m, 2H), 7.04–7.09 (m, 1H), 6.60 (br s, 1H), 2.39 (s, 3H); m/z (ES Mass) 377 (M+1, 100%); IR (cm^−1^, KBr) 3254, 1478, 1337, 1167.

### General method for the preparation of 2-substituted indoles **3**

A mixture of **1c** (300 mg, 0.95 mmol), 10% Pd/C (31.28 mg, 0.029 mmol), PPh_3_ (29.9 mg, 0.11 mmol), CuI (10.87 mg, 0.057 mmol) and 2-aminoethanol (2.85 mmol) in H_2_O (8 mL) was stirred at 25 °C for 1 h under nitrogen. The acetylenic compound **2** (2.85 mmol) was added slowly to the mixture with stirring. The reaction mixture was then stirred at 80 °C for the time indicated in Table 1. The mixture was cooled to room temperature, diluted with EtOAc (120 mL) and filtered through celite. The filtrate was collected, washed with cold water (2 × 75 mL), dried over Na_2_SO_4_, filtered and concentrated under vacuum. The residue thus obtained was purified by column chromatography (hexane/EtOAc) to afford the desired product.

Preparation of 2-(4-nitro-phenyl)-ethanesulfonic acid (**5**): A mixture of 2-(4-nitro-phenyl)ethylbromide (2.5 g, 11.0 mmol), sodium sulfite (2.05 g, 16.0 mmol) and TBAB (0.116 g, 0.36 mmol) in water was refluxed for 3 h. The reaction mass was cooled to room temperature and washed with ethyl acetate (5 mL). The aqueous layer was acidified (pH ~ 2) with dil HCl and concentrated. The gummy residue obtained was treated with methanol (10 mL) and stirred for 0.5 h. It was then filtered and the filtrate concentrated to give the crude product (2.4 g, 95% yield); brown liquid, *R*_f_ (80% ethyl acetate/*n*-hexane) 0.3; ^1^H NMR (DMSO-*d_6_*, 400 MHz) δ 8.15 (d, *J* = 8.4 Hz, 2H), 7.30 (d, *J* = 8.4 Hz, 2H), 3.21 (bs, OH, 1H), IR (cm^−1^, KBr): 3411, 1312, 1145; m/z (ESMS) 232 (M+1, 100%); ^13^C NMR (DMSO-*d_6_*, 50 MHz) δ 146.5, 145.2, 128.2 (2C), 123.5 (2C), 58.5, 29.5; HRMS (ESI): calcd for C_8_H_10_NO_5_S (M+H)^+^ 232.0278, found 232.0275.

Preparation of 2-(4-nitro-phenyl)ethanesulfonic acid methylamide (**7**): Compound **5** (3.70 g, 16.0 mmol) was cooled and PCl_5_ (8.30 g, 40.0 mmol) was added under anhydrous conditions. The mixture was slowly heated to reflux with stirring for 30 min. The reaction mixture was cooled to room temperature, diluted with CH_2_Cl_2_ (8 mL), stirred for 15 min and filtered. The filtrate containing the acid chloride **6** was collected and used in the next step directly. A saturated solution of methylamine was cooled to 0–5 °C and to this was added the CH_2_Cl_2_ solution of acid chloride **6** with stirring maintaining the temperature below 0 °C. The reaction mixture was stirred at the same temperature for 0.5 h and then at room temperature for 3 h. After completion of the reaction the mixture was filtered and the filtrate was collected and concentrated. The gummy residue obtained was stirred with cold water (100–150 mL) for 0.5 h and then filtered. The solid obtained was washed for several times with water, 20% ethyl acetate/*n*-hexane and dried to give the title compound crude product (1.5 g, 38% yield) as a brown solid; mp 132–136 °C; *R*_f_ (60% ethyl acetate/*n*-hexane) 0.3; ^1^H NMR (DMSO-*d_6_*, 400 MHz) 8.17 (d, *J* = 8.4 Hz, 2H), 7.40 (d, *J* = 8.4 Hz, 2H), 4.12 (bs, NH, 1H), 3.27–3.22 (m, 2 H), 2.87–2.83 (m, 2H), 2.70 (d, *J* = 4.5 Hz, 3H); IR (cm^−1^, KBr) 3411, 1312, 1145; m/z (ES Mass) 245 (M+1, 100%); ^13^C NMR (DMSO-*d_6_*, 50 MHz) δ 148.5, 145.6, 128.7 (2C), 124.5 (2C), 58.5, 24.8, 24.4; HRMS (ESI): calcd for C_9_H_13_N_2_O_4_S (M+H)^+^ 245.0595, found 245.0592.

Preparation of 2-(4-amino-phenyl)ethanesulfonic acid methylamide (**8**): A solution of compound **7** (1.55 g, 6.3 mmol) was dissolved in 1:1 methanol/ethyl acetate (30 mL) and hydrogenated in the presence of Raney nickel (1.5 g) at 65 to 70 psi for 4–5 h. The reaction mixture was filtered through celite bed. The filtrate was collected, concentrated and dried to give the title compound (1.0 g, 74% yield) as a brown solid; mp 126–128 °C; *R*_f_ (75% ethyl acetate/*n*-hexane) 0.3; ^1^H NMR (DMSO-*d_6_* 400 MHz) δ 6.90 (d, *J* = 8.5 Hz, 2H), 6.88 (bs, NH, 1H), 6.50 (d, *J* = 8.2 Hz, 2H), 4.91 (bs, NH, 2H), 3.17–3.12 (m, 2 H), 2.77–2.73 (m, 2H), 2.56 (d, *J* = 4.5 Hz, 3H); IR (KBr, cm^−1^) 3411, 1312, 1145; m/z (ES Mass): 215 (M+1, 100%); ^13^C NMR (DMSO-*d_6_*, 50 MHz): 146.9, 128.7 (2C), 125.2, 114.0 (2C), 51.0, 28.5, 28.3; HRMS (ESI): calcd for C_9_H_15_N_2_O_2_S (M+H)^+^ 215.0854, found 215.0856.

Preparation of 2-(4-amino-3-iodo-phenyl)ethanesulfonic acid methylamide (**9**): A suspension of compound **8** (1.0 g, 4.67 mmol) in aqueous solution (10.0 mL) of sodium bicarbonate (4.67 mmol) was stirred for 0.5 h. Then iodine (4.20 mmol) was added at 5–10 °C and the mixture was stirred for 6.0 h. After completion, the reaction mixture was diluted with ethyl acetate (50 mL) and extracted with ethyl acetate (3 × 50 mL). The organic layers were collected, combined, washed with saturated aqueous sodium thiosulfate solution (2 × 25 mL), dried over anhydrous Na_2_SO_4_ and concentrated under vacuum. The crude product was purified by column chromatography on silica gel using 7:3 hexane/ethyl acetate to afford the desired product (1.24 g, 78% yield); brown solid; mp 98–100 °C; *R*_f_ (60% ethyl acetate/*n*-hexane) 0.3; ^1^H NMR (CDCl_3_, 400 MHz) δ 7.41 (s, 1H), 6.98 (d, *J* = 8.0 Hz, 1H), 6.89 (bs, NH, 1H), 6.69 (d, *J* = 8.0 Hz, 1H), 5.06 (bs, NH, 2H), 3.19–3.15 (m, 2 H), 2.74–2.73 (m, 2H), 2.58 (d, *J* = 4.5 Hz, 3H); IR (cm^−1^, KBr) 3387, 1499, 1307, 1147; m/z (ES Mass) 341 (M+1, 100%); ^13^C NMR (DMSO-*d_6_*, 200 MHz): 146.8, 137.8, 129.1, 128.0, 114.2, 83.1, 50.6, 28.5, 27.6; HRMS (ESI): calcd for C_9_H_14_N_2_O_2_SI (M+H)^+^ 340.9821, found 340.9824.

Preparation of 2-(3-iodo-4-methanesulfonylamino-phenyl)ethanesulfonic acid methylamide (**10**): A mixture of compound **9** (1.0 g, 2.94 mmol), DMAP (53.21 mg, 0.43 mmol) was dissolved in pyridine (5.0 mL) and cooled to 0–5 °C. To this was added methanesulfonyl chloride (3.82 mmol) dropwise and the mixture was refluxed for 12 h. After completion of the reaction, the mixture was cooled to 5–10 °C, neutralized with dil HCl and extracted with ethyl acetate (3 × 50 mL). The organic layers were collected, combined, washed with saturated aq. NaCl (2 × 25 mL), dried over anhydrous Na_2_SO_4_ and concentrated under vacuum. The crude compound was purified by column chromatography on silica gel using 9:1 hexane/ethyl acetate to afford the desired product (1.0 g, 82% yield) as a brown solid, mp 156–158 °C; *R*_f_ (20% ethyl acetate/*n*-hexane) 0.3; ^1^H NMR (DMSO-*d_6_*, 400 MHz) δ 7.88 (s, 1H), 7.40–7.34 (m, 3H), 6.57 (bs, NH, 1H ), 3.26–3.23 (m, 2H), 3.21–3.02 (m, 2H), 2.81 (s, 3H), 2.71 (d, *J* = 4.5 Hz, 3H); IR (cm^−1^, KBr) 3276, 1476, 1364, 1156; m/z (ES Mass): 418 (M+1, 100%); ^13^C NMR (DMSO-*d_6_*, 200 MHz): 142.8, 140.1, 135.0, 131.7, 129.6, 104.0, 49.5, 43.8, 28.5, 28.1; HRMS (ESI): calcd for C_10_H_16_N_2_O_4_S_2_I (M+H)^+^ 418.9596, found 418.9607.

Preparation of 2-(1-methanesulfonyl-2-phenyl-1*H*-indol-5-yl)ethanesulfonic acid methylamide (**C**): A mixture of **10** (1.0 g, 2.39 mmol), 10% Pd/C (76.0 mg, 0.071 mmol), PPh_3_ (75.22 mg, 0.28 mmol), CuI (27.4 mg, 0.14 mmol) and 2-aminoethanol (0.44 g, 7.17 mmol) in H_2_O (25 mL) was stirred at 25 °C for 1 h under nitrogen. The acetylenic compound **2g** (0.73 g, 7.17 mmol) was added slowly to the mixture with stirring. The reaction mixture was then stirred at 80 °C for 12 h. The mixture was cooled to room temperature, diluted with EtOAc (250 mL) and filtered through celite. The filtrate was collected, washed with cold water (2 × 125 mL), dried over Na_2_SO_4_, filtered and concentrated under vacuum. The residue thus obtained was purified by column chromatography (hexane/EtOAc) to afford the desired product (0.75 g, 80% yield) as a brown solid, mp 150–152 °C; *R*_f_ (60% ethyl acetate/*n*-hexane) 0.3; ^1^H NMR (DMSO-*d_6_*, 400 MHz) δ 7.89 (d, *J* = 8.2 Hz, 2H), 7.58–7.51 (m, 3H), 7.43–7.31 (m, 3H), 6.99 (bs, NH, 1H), 6.81 (s, 1H), 3.35–3.29 (m, 2H), 3.08–3.04 (m, 5H), 2.60 (d, *J* = 4.8 Hz, 3H); IR (cm^−1^, KBr) 3275, 1363, 1320, 1173.6; m/z (ES Mass) 393 (M+1, 100%); ^13^C NMR (DMSO-*d_6_*, 200 MHz) δ 141.5, 135.9, 134.4, 131.9, 129.9, 129.8 (2C), 127.5 (2C), 125.3, 120.5, 115.0, 112.0, 59.7, 50.7, 38.6, 30.6, 20.7; HRMS (ESI): calcd for C_18_H_21_N_2_O_4_S_2_ (M+H)^+^ 393.0943, found 393.0942.

## Supporting Information

File 1 Spectral data of 2-substituted indoles **3a–s.**

## References

[1] Holenz J, Pauwels P J, Díaz J L, Mercè R, Codony X, Buschmann H (2006). Drug Discovery Today.

[2] Riggs J R, Kolesnikov A, Hendrix J, Young W B, Shrader W D, Vijaykumar D, Stephens R, Liu L, Pan L, Mordenti J (2006). Bioorg Med Chem Lett.

[3] Kolesnikov A, Rai R, Young W B, Mordenti J, Liu L, Torkelson S, Shrader W D, Leahy E M, Hu H, Gjerstad E (2006). Bioorg Med Chem Lett.

[4] Sugimoto Y, Shimizu A, Kato T, Satoh A, Ozaki S, Ohta H, Okamoto O (2006). Bioorg Med Chem Lett.

[5] Koppitz M, Reinhardt G, van Lingen A (2005). Tetrahedron Lett.

[6] Payack J F, Vazquez E, Matty L, Kress M H, McNamara J (2005). J Org Chem.

[7] Brands M, Ergüden J-K, Hashimoto K, Heimbach D, Schröder C, Siegel S, Stasch J-P, Weigand S (2005). Bioorg Med Chem Lett.

[8] Russell M G N, Matassa V G, Pengilley R R, van Niel M B, Sohal B, Watt A P, Hitzel L, Beer M S, Stanton J A, Broughton H B (1999). J Med Chem.

[9] Humphrey G R, Kuethe J T (2006). Chem Rev.

[10] Krüger K, Tillack A, Beller M (2008). Adv Synth Catal.

[11] Ackermann L (2007). Synlett.

[12] Joucla L, Djakovitch L (2009). Adv Synth Catal.

[13] Cacchi S, Fabrizi G (2005). Chem Rev.

[14] Pal M, Subramanian V, Batchu V R, Dager I (2004). Synlett.

[15] Dains F B Y, Vaughan T H, Janney W M (1918). J Am Chem Soc.

[16] Rosauer K G, Ogawa A K, Willoughby C A, Ellsworth K P, Geissler W M, Myers R W, Deng Q, Chapman K T, Harris G, Molle D E (2003). Bioorg Med Chem Lett.

[17] Stefano S, Claudio F (2005). Synlett.

[18] James C L, James J F, George F A, Amy M, Do W H, Zhihua S J (2006). Bioorg Med Chem Lett.

