# Supplementary material for: Pd/C-Mediated synthesis of indoles in water
Source: Beilstein J Org Chem. 2009 Sep 23;5:46. doi: 10.3762/bjoc.5.46 (PMC2779661; doi:10.3762/bjoc.5.46)
Supplement: File 1 — Spectral data of 2-substituted indoles 3a–s. [file Beilstein_J_Org_Chem-05-46-s001.doc]

Supporting Information

## Pd/C-Mediated synthesis of indoles in water

Mohosin Layek^1,2^, Udaya Lakshmi^1^, Dipak Kalita^1^, Deepak Kumar Barange^1^, Aminul Islam^1^, K. Mukkanti^2^, Manojit Pal^3^,*

Address:

^1^Dr. Reddy’s Laboratories Ltd, Bollaram Road, Miyapur, Hyderabad 500049, India. ^2^Chemistry Division, Institute of Science and Technology, JNT University, Kukatpally, Hyderabad 500072, India and ^3^New Drug Discovery, R&D Center, Matrix Laboratories Ltd., Anrich Industrial Estate, Bollaram, Jinnaram Mandal, Medak District, Andra Pradesh 502 325, India

Email: manojitpal@rediffmail.com

*Corresponding author

**Spectral data of 2-substituted indoles 3a-s**

**1-(5-Chloro-1-methanesulfonyl-1*H*-indol-2-yl)-propan-2-ol (3a):** white solid, mp 45-46 °C; R*_f_* (25 % ethyl acetate/ n-hexane) 0.3; ^l^H NMR (CDCl_3_, 400 MHz) δ 7.93 ( d, *J* = 8.5 Hz, 1H), 7.49 (s, 1H), 7.26 (d, *J* = 8.5 Hz, 1H), 6.53 (s, 1H), 4.19-3.95 (m, 1H), 3.04 (s, 3H), 2.45 (d, *J* = 6.5 Hz, 2H), 1.32 (bs, OH), 1.28 (d, *J* = 6.5 Hz, 3H); IR (cm^-1^, Neat) 3356, 1448, 1365, 1169; m/z (ES Mass) 288 (M+1, 100%); ^13^C NMR (CDCl_3_, 50 MHz) δ 140.0, 153.1, 130.8, 129.5, 124.3, 120.0, 115.2, 110.0, 74.3, 40.6, 38.2, 23.1; HPLC: 99.8 %, column: Inertsil ODS 3V (150 x 4.6) mm, mobile Phase A: 0.01 M KH_2_PO_4_ (pH 3.0 with H_3_PO_4_), mobile Phase B: CH_3_CN; gradient elution 0/60, 1/60, 6/80, 16/80, 19/60, 20/60, flow rate: 1.5 mL/min, UV: 226 nm, retention time 8.1 min; Elemental Analysis found: C, 50.10; H, 4.80; N, 4.90 C_12_H_14_ClNO_3_S, Requires: C, 50.09; H, 4.90; N, 4.87.

**5-Chloro-1-methanesulfonyl-2-p-tolyl-1*H*-indole (3b):** white solid, mp 207-209 °C; R***_f_*** (20% ethyl acetate/ n-hexane) 0.3; ^l^H NMR (CDCl_3_, 400 MHz) δ 7.90 (d, *J* = 8.0 Hz, 2H), 7.40 (s, 1H), 7.30 (d, *J* = 8.0 Hz,1H), 7.15 (d, *J* = 6.0 Hz, 1H), 7.09 (t, *J* = 8.0 Hz, 2H), 6.46 (s, 1H), 2.59 (s, 3H), 2.27 (s, 3H); IR (cm^-1^, KBr) 1445, 1353, 1172; m/z (ES Mass) 320 (M+1, 100 %); ^13^C NMR (CDCl_3_, 50 MHz) δ 143.46, 139.3, 131.4, 130.1, 128.5 (2C), 125.0 (2C), 120.4 (2C), 116.9 (2C), 111.7 (2C), 39.8, 21.4; HPLC: 99.7%, Column: Inertsil ODS 3V (150 x 4.6) mm, mobile phase A: 0.01M KH_2_PO_4_, mobile phase B: CH_3_CN Gradient elution (T/ % B); 0/60, 2/60, 8/80, 18/80, 20/60, 22/60; flow Rate: 1.5 mL/min, UV: 210 nm, Retention Time 9.2 min Elemental Analysis found: C, 59.84; H, 4.29; N, 4.49 C_16_H_14_ClNO_2_S Requires: C, 60.09; H, 4.41; N, 4.38.

**5-Ethyl-1-methanesulfonyl-2-p-tolyl-1*H*-indole (3c):** white solid, mp 125-130 °C; R***_f_*** (20% ethyl acetate/ n-hexane) 0.3; ^l^H NMR (CDCl_3_, 400 MHz) δ 8.01 (d, *J* = 8.0 Hz, 1H), 7.45 (d, *J* = 8.2 Hz, 1H), 7.43 (d, *J* = 8.2 Hz, 1H), 7.25 (s, 1H), 7.23-7.19 (s, 3H), 6.62 (s, 1H), 2.78-2.73 (q, *J* = 7.5, 2H), 2.69 (s, 3H), 2.41 (s, 3H), 1.31-1.28 (t, *J* = 8.5 Hz, 3H); IR (cm^-1^, KBr) 1360, 1168, 816; m/z (ES Mass) 314 (M+1, 100%); ^13^C NMR (CDCl_3_, 50 MHz) δ 142.7, 140.6, 138.6, 136.2, 130.5, 129.8, 129.4, 128.3, 127.4, 127.4, 125.1, 119.5, 115.6, 112.6, 39.0, 28.6, 21.3, 51.9 ; HPLC: 99.4 %, Column: Inertsil ODS 3V (150 x 4.6) mm, mobile phase A: 0.01 M KH_2_PO_4_ (pH 6.5), mobile phase B; CH_3_CN, gradient elution : 0/60, 1/60, 6/80, 16/80, 19/60, 20/60; Flow Rate: 1.5 mL/min, UV: 210 nm, retention time 9.2 min; Elemental Analysis found: C, 68.94; H, 5.90; N, 4.35 C_18_ H_19_NO_2_S Requires: C, 68.98; H, 6.11; N, 4.4.

**5-Ethyl-2-(indol-1-ylmethyl)-1-methanesulfonyl-1*H*-indole (3d):** light brown solid, mp 120-125 °C; R***_f_*** (20% ethyl acetate/ n-hexane) 0.32; ^l^H NMR (CDCl_3_, 400 MHz) δ 7.86 (d, *J* = 8.0 Hz, 1H), 7.64 (d, *J* = 8.0 Hz, 1H**),** 7.55 (d, *J* = 8. 1Hz, 1H), 7.20 (s, 1H), 7.19 (d, *J* = 8.0 Hz, 1H), 7.16-7.10 (m, 3H), 6.56 (s, 1H), 6.23 (s, 1H), 5.64 ( s, 2H), 2.77 (s, 3H), 2.72 (q, *J* = 7.4 Hz, 2H), 1.30 (t, *J* = 7.4 Hz, 3H); IR (cm^-1^, KBr) 1365, 1234, 1165, 745; m/z (ES Mass) 353 (M+1, 100 %); ^13^C NMR (CDCl_3_, 50 MHz) δ 139.8, 136.1, 136.1, 135.3, 128.9, 128.6, 128.2, 121.9, 121.7, 120.9, 119.8, 113.5, 110.8, 109.8, 102.0, 62.9, 44.3, 40.3, 29.6, 15.9; HPLC: 99.9% Column: Inertsil ODS 3V (150 x 4.6) mm, mobile phase A: 0.01M KH_2_PO_4_ (pH 3.0 with H_3_PO_4_), mobile Phase B; CH_3_CN, gradient elution (T/ % B); 0/60, 1/60, 6/80, 16/80, 19/60, 20/60, Flow Rate: 1.5 mL/min, UV: 226 nm, Retention Time 7.5 min; Elemental Analysis found: C, 69.98; H, 5.58; N, 8.05 C_20_H_20_N_2_O_2_S Requires: C, 68.16; H, 5.72; N, 7.95.

**5-Fluoro-1-methanesulfonyl-2-p-tolyl-1*H*-indole (3e):** white solid, mp 209-210 °C; R***_f_*** (20% ethyl acetate/ n-hexane) 0.35; ^l^H NMR (CDCl_3_, 400 MHz) δ 8.08-8.05 (m, 1H), 7.45 (d, *J* = 8.2 Hz, 1H), 7.43 (d, 8.2 Hz, 1H), 7.25-7.21 (m, 3H), 7.09 (d, *J* = 8.0, 1H), 6.63 (s, 1H), 2.72 (s, 3H), 2.41 (s, 3H); IR (cm^-1^, KBr) 1464, 1360, 1169; m/z (ES Mass) 304 (M+1, 100 %) ^13^C NMR (CDCl_3_, 50 MHz) δ 162.6, 157.8, 143.8, 139.2, 134.1, 131.4, 131.2, 128.5, 117.1, 112.4, 112.3, 112.2, 106.6, 106.1, 39.4, 21.3; HPLC 99.6 % Column: Symmetry Shield RP 8 (150 x 4.6) mm, mobile phase A: 0.01 M KH_2_PO_4_ (pH 3.0), mobile phase B: CH_3_CN; gradient elution (T /% B): 0/60, 3/60, 8/80, 18/80, 19/60, 20/60; flow rate: 1.5 ml/min., UV: 210 nm, retention time 5.9, elemental analysis found: C, 63.24; H, 4.61; N, 4.49 C_16_H_14_FNO_2_S Requires: C, 63.34; H, 4.65; N, 4.61.

**2-(5-Benzyloxy-indol-1-ylmethyl)-5-fluoro-1-methanesulfonyl-1*H*-indole (3f):** white solid, mp 144-145; R***_f_*** (20% ethyl acetate/ n-hexane) 0.25; ^1^H NMR (CDCl_3_, 400 MHz) 7.92-7.89 (m, 1H), 7.47 (d, *J* = 7.8 Hz, 1H), 7.45 (d, *J* = 7.8 Hz, 1H), 7.39 (s, 1H), 7.37 (d, *J* = 7.8 Hz, 1H), 7.36 (d, *J* = 8.0 Hz, 1H), 7.32 (s,1H), 7.31 (d, *J* = 7.8 Hz, 1H), 7.25 (d, *J* = 7.9 Hz, 1H), 7.17 (t, *J* = 8.0 Hz, 2H), 6.94 (t, *J* = 8.0 Hz, 1H), 6.48 (d, *J* = 7.8 Hz, 1H), 6.42 (s, 1H), 5.59 (s, 2H), 5.09 (s,2H), 2.78 (s, 3H); IR (cm^-1^, KBr) 1487.3, 1364.7, 1234.3, 1168.3; m/z (ES Mass) 449 (M+1, 100 %); ^13^C NMR (CDCl_3_, 50 MHz) 162.0, 157.2, 153.5, 137.9, 137.5, 133.1, 131.5, 129.7, 129.5, 129.0, 128.8, 128.4, 127.7, 127.4, 114.7, 112.9, 110.5, 109.9, 106.8, 106.3, 104.2, 101.9, 70.7, 44.5, 40.6; HPLC: 98.5 % Column: Inertsil ODS 3V (150 x 4.6) mm, mobile Phase A: 0.01M KH_2_PO_4_, mobile Phase B; CH_3_CN, gradient elution; 0/65, 2/65, 6/80, 20/80, 24/65, 25/65, flow rate: 1.5 mL/min, UV 210 nm, retention time 7.9 min; Elemental Analysis found: C, 68.20; H, 4.72; N, 6.05, C_25_H_21_FN_2_O_3_S Requires: C, 66.94; H, 4.71; N, 6.24.

**5-Fluoro-2-(indol-1-ylmethyl)-1-(toluene-4-sulfonyl)-1*H*-indole (3g):** off white solid, mp 112-114 °C; R***_f_*** (25% ethyl acetate/ n-hexane) 0.27; ^1^H NMR (CDCl_3_, 400 MHz) δ 8.09 (d, *J* = 8.2 Hz, 1H), 7.64 (d, *J* = 8.0 Hz, 1H), 7.63 (d, *J* = 8.0 Hz, 1H), 7.61 (d, *J* = 8.2 Hz, 1H), 7.25-722 (m, 2H), 7.14-7.11 (m, 2H), 7.09-7.00 (m, 2H), 6.98 (d, *J* = 8.2 Hz, 1H), 6.92 (d, *J* = 8.5 Hz, 1H), 6.56 (s, 1H), 5.77 (s, 1H), 5.67 (s, 2H), 2.38 (s, 3H); IR (cm^-1^, CHCl_3_**)** 1464, 1376, 1169; m/z (ES Mass) 419 (M+1, 100%); ^13^C NMR (CDCl_3_, 50 MHz) δ 162.1, 157.3, 145.4, 138.5, 136.1, 135.4, 133.6, 130.1, 128.6, 128.3, 126.2, 121.9, 121.0, 119.8, 115.5, 115.3, 112.7, 112.2, 109.8, 109.7, 106.5, 102.3, 44.9, 21.5; HPLC: 98.1% Symmetry Shield RP 8 (150 x 4.6) mm, mobile phase A: 0.01 M KH_2_PO_4_ (pH 6.5 with KOH), mobile Phase B: CH_3_CN gradient elution (T /% B): 0/60, 1/60, 6/80, 18/80, 19/60, 20/60 flow rate: 1.5 mL/min., UV: 220 nm, retention time 7.5 min; Elemental Analysis found: C, 69.05; H, 4.32; N, 6.55 C_24_H_19_FN_2_O_2_S Requires: C, 68.88; H, 4.58; N, 6.69.

**3-[5-Fluoro-1-(toluene-4-sulfonyl)-1*H*-indol-2-yl]-propan-1-ol (3h):** off white solid, mp 88-90 °C; R***_f_*** ( 25% ethyl acetate/ n-hexane) 0.3; ^1^H NMR (CDCl_3_, 400 MHz) δ 8.10 (d, *J* = 8.2 Hz, 1H), 7.59 (d, *J* = 8.0 Hz, 1H), 7.57 (d, *J* = 8.2 Hz , 1H), 7.19 ( d, *J* = 8.2 Hz ,1H), 7.17 (d, *J* = 8.0 Hz, 1H), 7.05 (d, *J* = 8.5 Hz, 1H), 7.03 (s, 1 H), 6.37 (s, 1H), 3.74 (t, *J* = 6.0 Hz, 2H), 3.11-3.07 (m, 2H), 2.33 (s, 3H), 2.16-2.20 (m, 2H), 1.25 (bs, OH); IR cm^-1^ (CHCl_3_) 1469, 1368, 1176; m/z (ES Mass) 348 (M+1, 100 %); ^13^C NMR (CDCl_3_, 50 MHz) δ 162.1, 157.3, 144.9, 143.4, 135.6, 133.4, 130.9, 129.8, 126.2, 115.9, 111.8, 111.3, 109.1, 105.5, 61.8, 32.1, 25.5, 21.5; HPLC 98.7 %, Column: Symmetry Shield RP 8 (150 x 4.6) mm, mobile Phase A: 0.01 M KH_2_PO_4_ (pH 6.5 with KOH), mobile Phase B: CH_3_CN; gradient elution (T /% B): 0/40, 2/40, 10/80, 18/80, 19/60, 20/40 Flow rate: 1.5 mL/min., UV: 220 nm, retention time 8.1 min; Elemental Analysis found: C, 61.98; H, 5.07; N, 4.18 C_18_H_18_FNO_3_S Requires: C, 62.23; H, 5.22; N, 4.03.

**1-[5-Fluoro-1-(toluene-4-sulfonyl)-1*H*-indol-2-yl]-propan-2-ol (3i):** mp > 20 °C; R***_f_*** (25 % ethyl acetate/ n-hexane) 0.27; ^1^HMR (CDCl_3_, 400 MHz) δ 8.10 (d, *J* = 8.0 Hz, 1H), 7.58 (d, *J* = 8.2 Hz, 1H), 7.56 (d, *J* = 8.4 Hz, 1H), 7.25-7.17 (m, 2H), 7.08 (d, *J* = 8.0 Hz, 1H), 6.99 (s, 1H), 6.45 (s, 1H), 4.26-4.25 (m, 1H), 3.25-3.01 (m, 2H), 2.33 (s, 3H), 1.32 (bs, OH), 1.31 (d, *J* = 6.5 Hz, 2H) IR (cm^-1^, CHCl_3_) 1467, 1365, 1173; m/z (CI Mass): 348 (M+1, 100 %) ;^13^C NMR (CDCl_3_, 50 MHz) δ 162.1, 157.3, 145.0, 140.3, 135.4, 133.5, 130.8, 130.6**,** 129.8, 126.1, 115.9, 111.1, 111.0, 106.0, 67.1, 38.9, 22.9, 21.4; HPLC: 99.7 %; Column: Inertsil ODS 3V (150 x 4.6) mm, mobile Phase A: 0.01M KH_2_PO_4_,  mobile phase B; CH_3_CN Gradient elution (T/ % B); 0/60, 2/60, 8/80, 18/80, 20/60, 22/60, flow rate: 1.5 mL/min, UV: 225 nm, retention time 8.2 min; Elemental Analysis found: C, 62.06; H, 5.34; N, 4.23 C_18_H_18_FNO_3_S Requires: C, 62.23; H, 5.22; N, 4.03.

**2-[5-Fluoro-1-(toluene-4-sulfonyl)-1*H*-indol-2-yl]-propan-2-ol (3j):** pale yellow low melting solid; R***_f_*** (25% ethyl acetate/ n-hexane) 0.25; ^1^H NMR (CDCl_3_, 400 MHz) δ 7.95 (d, *J* = 8.0 Hz, 1H), 7.69 (d, *J* = 8.0 Hz, 1H), 7.67 (d, *J* = 8.0 Hz, 1H), 7.18 (s, 1H), 7.16 (d, *J* = 8.0 Hz, 1H), 7.04 (d, 1H), 6.9 (m, 1H), 6.64 (s, 1H), 2.30 (s, 3H), 1.82 (s, 6H), 1.33 (bs, OH); IR (cm^-1^, CHCl_3_) 1598, 1460, 1366, 1174 m/z (CI Mass) 348 (M+1, 100 %); ^13^C NMR (CDCl_3_, 50 MHz, CDCl_3_) δ 162.1, 157.3, 150.0, 144.9, 134.6, 129.5, 129.5, 126.4, 126.4, 116.7, 116.5, 112.9, 112. 4, 106.3, 69.2, 30.8, 30.2, 21.3; HPLC: 98.9 % Column: Inertsil ODS 3V (150 x 4.6) mm, mobile phase A: 0.01M KH_2_PO_4_, mobile phase B: CH_3_CN; gradient elution; 0/50, 3/50, 8/80, 18/80, 20/50, 50/50, Flow Rate: 1.5 mL/min, UV: 217 nm, retention time 9.3 min; Elemental Analysis found: C, 61.96; H, 5.06; N, 4.15 C_18_H_18_FNO_3_S Requires: C, 62.23; H, 5.22; N, 4.03.

**5-Fluoro-2-(indol-1-ylmethyl)-1-methanesulfonyl-1*H*-indole (3k):** pale yellow solid, mp 112-114 °C ; R***_f_*** (30% ethyl acetate/ n-hexane) 0.25; ^1^H NMR (CDCl_3_, 400 MHz) δ 7.91 (t, *J* = 8.1 Hz, 1H), 7.64 (d, *J* = 8.0 Hz, 1H), 7.34 (d, *J* = 8.2 Hz, 1H), 7.25 (s, 1H), 7.20 (d, *J* = 8 .0 Hz, 1H), 7.16 (d, *J* = 8.5 Hz, 1H), 7.13 (d, *J* = 8.0 Hz, 1H), 7.11 (t, *J* = 7.0 Hz, 1H), 6.58 (d, *J* = 7.5 Hz, 1H), 6.21 (s, 1H), 5.64 (s, 2H), 2.81 (s, 3H); IR (cm^-1^, CHCl_3_) 1613, 1465, 1161; m/z (CI Mass) 343 (M+1, 100%) ^13^C NMR (50 MHz, CDCl_3_) δ 162.1, 157.3, 137.9, 136.1, 133.1, 129.7, 122.0, 121.1, 119.9, 114.7, 113.1, 110.5, 110.4, 109.7, 106.8, 102.3, 44.4, 40.7; HPLC 97.8%, Column: Inertsil ODS 3V (150 x 4.6) mm, mobile phase A: 0.01M KH_2_PO_4_, mobile phase B: CH_3_CN; gradient elution 0/50, 3/50, 8/80, 18/80, 20/50, 22/50, Flow Rate: 1.5 ml/min, UV: 217 nm, retention time 9.0 min; Elemental Analysis found: C, 62.97; H, 4.32; N, 8.11 C_18_H_15_FN_2_O_2_S Requires: C, 63.14; H, 4.42; N, 8.18.

**5-Fluoro-1-(toluene-4-sulfonyl)-2-p-tolyl-1*H*-indole (3l):** Off white solid, mp 136-138 °C; R***_f_*** (30% ethyl acetate / n-hexane) 0.25; ^1^H NMR (CDCl_3_, 400 MHz) δ 8.25-8.22 (m, 1H), 7.39-7.22 (m, 6H), 7.07 (m, 4H), 6.45 (s, 1H) 2.43 (s, 3H), 2.29 (s, 3H); IR (cm^-1^, CHCl_3_) 1458, 1369, 1199; Mass (CI Mass) 380 (M+1, 100 %); ^13^C NMR (50 MHz, CDCl_3_) δ 162.5, 157.7, 144.6, 144.1, 138.8, 134.4, 131.8, 131.6, 130.0, 130.0 (2C), 129.1(2C), 128.2 (2C), 126.7, 117.8, 112.9, 112.4, 106.3, 21.3, 21.3; HPLC 99.2 %, Column: Symmetry Shield RP 8 (150 x 4.6) mm, mobile phase A: 0.01 M KH_2_PO_4_ (pH 6.5 with KOH), mobile Phase B: CH_3_CN; gradient elution T % B: 0/60, 1/60, 6/80, 18/80, 19/60, 20/60, flow rate: 1.5 ml/min., UV: 210 nm, retention time 6.9 min; Elemental Analysis found: C, 69.47; H, 4.82; N, 3.79 C_22_H_18_FNO_2_S Requires: C, 69.64; H, 4.78; N, 3.69.

**5-Fluoro-2-phenyl-1-(toluene-4-sulfonyl)-1*H*-indole (3m):** off white solid, mp 110-112 °C; R***_f_*** (20% ethyl acetate/ n-hexane) 0.25; ^1^H NMR (CDCl_3_, 400 MHz) δ 8.27 (d, *J* = 8.5 Hz, 1H), 7.47 (d, *J* = 8.2 Hz, 1H), 7.43 (d, *J* = 8.4 Hz, 1H), 7.42 (d, *J* = 8.4 Hz, 1H), 7.25 (d, *J* = 8.0 Hz, 1H), 7.24 ( d, *J* = 8.0 Hz, 1H), 7.22 (m, 2H), 7.09 (d, *J* = 8.2 Hz, 1H), 7.04 (d, *J* = 8.5 Hz, 2H), 7.03 (s, 1H), 6.49 (s, 1H), 2.29 (s, 3H); IR (cm^-1^, CHCl_3_) 1460, 1378, 1173; Mass (CI Mass) 366 (M+1, 100 %); ^13^C NMR (50 MHz, CDCl_3_) δ 162.5, 157.6, 144.7, 143.9, 134.3, 131.9, 131.7, 131.5, 130.2 (2C), 129.2 (2C), 128.8 (2C), 127.5, 126.7, 117.7, 113.2, 112.2, 106.4, 21.4; HPLC: 99.1 % Column: Symmetry Shield RP 8 (150 x 4.6) mm, mobile phase A: 0.01 M KH_2_PO_4_, mobile phase B: CH_3_CN, gradient elution T % B: 0/60, 2/60, 7/80, 17/80, 19/60, 20/60 Flow rate: 1.5 ml/min, UV: 210 nm, retention time 7.2 min; Elemental Analysis Found : C, 68.88 ; H, 4.23; N, 3.84 C_21_H_16_FNO_2_S Requires: C, 69.02; H, 4.41; N, 3.83.

**2-[5-Fluoro-1-(toluene-4-sulfonyl)-1*H*-indol-2-ylmethyl]-isoindole-1,3-dione (3n):** white solid, mp 182-184 °C; R***_f_*** (20% ethyl acetate/ n-hexane) 0.25; ^1^H NMR (CDCl_3_, 400 MHz) δ 8.02 (d, *J* = 8.2 Hz, 1H), 7.94 (d, *J* = 8.0 Hz, 2H), 7.92 (d, *J* = 8.0 Hz, 3H), 7.38 –7.25 (m, 3H), 6.99- 6.94 (m, 2H, 6.20 (s, 1H), 5.35 (s, 2H), 2.36 (s, 3H); IR (cm^-1^, CHCl_3_) 1710, 1381, 1164; m/z (CI Mass) 449 (M+1, 100 %); ^13^C NMR (50 MHz, CDCl_3_) δ 167.5, 162.0, 157.2, 145.2, 137.1, 134.9, 134.3, 134.0, 131.8, 130.0, 128.4, 128.2, 128.2, 126.5, 126.5, 123.6, 115.6, 112.5, 108.4, 106.3, 105.8, 15.8, 36.6, 21.0; HPLC: 97.5 % Column: Symmetry Shield RP 8 (150 x 4.6) mm, mobile phase A: 0.01 M KH_2_PO_4_, mobile phase B: CH_3_CN; gradient elution T % B: 0/60, 2/60, 7/80, 17/80, 19/60, 20/60; flow rate: 1.5 mL/min., UV: 220 nm, retention time 6.3 min, Elemental Analysis Found : C, 64.09; H, 3.90; N, 6.05 C_24_H_17_FN_2_O_4_S Requires : C, 64.28; H, 3.82; N, 6.25.

**2-Butyl-5-fluoro-1-(toluene-4-sulfonyl)-1*H*-indole (3o): w**hite solid, mp 102-104 °C; R***_f_*** (25% ethyl acetate/ n-hexane) 0.3; ^1^H NMR (CDCl_3_, 400 MHz) δ 8.10 (d, *J* = 8.5 Hz, 1H), 7.58 (d, *J* = 8.0 Hz, 2H), 7.18 (d, *J* = 8.0 Hz, 2H), 7.05 (d. *J* = 8.2 Hz, 1H), 6.96 (m, 1H), 6.33 (s, 1H), 2.96 (t, *J* = 7.5 Hz, 2H), 2.33 (s, 3H), 1.75-1.67 (m, 2H), 1.47-1.42 (m, 2H), 0.97-0.93 (t, *J* = 7.2 Hz, 3H); IR (cm^-1^, CHCl_3_): 2959, 1598, 1467, 1370, 1166; Mass (CI method): 346 (M+1) 100 %; ^13^C NMR (50 MHz, CDCl_3_) δ 162.1, 157.3, 144.7, 144.3 (2C), 136.0, 133.0 (2C), 131.0, 130.8, 115.7, 111.6, 108.5, 105.8, 64.8, 30.1, 29.6, 28.7, 22.4, 13.8; HPLC 97.7 %, Column Symmetry Shield RP 8 (150 x 4.6) mm, mobile Phase A: 0.01 M KH_2_PO_4_, mobile Phase B: CH_3_CN, Gradient elution T % B: 0/60, 1/60, 8/80, 16/80, 18/60, 20/60, flow rate: 1.5 ml/min., UV 220 nm, retention time 10.8 min; Elemental Analysis Found: C, 66.15; H, 5.72, N, 4.20 C_19_H_20_FNO_2_S Requires: C, 66.06; H, 5.84; N, 4.05.

**5-Fluoro-1-methanesulfonyl-2-(2-nitro-phenyl)-1*H*-indole (3p):** mp > 20 °C; R***_f_*** (30% ethyl acetate/ n-hexane) 0.3; ^1^H NMR (CDCl_3_, 400 MHz) 8.12 (d, *J* = 8.0 Hz, 1H), 7.98-7.94 (m, 2H), 7.78-7.56 (m, 2H), 7.37-7.36 (m, 1H), 7.15-7.12 (m, 1H), 6.55 (s, 1H), 3.11 (s, 3H); IR (cm^-1^, KBr) 3108, 1357, 1174; m/z (ES Mass) 335 (M+1, 100 %); ^13^C NMR (CDCl_3_, 50 MHz) δ 162.2, 157.4, 148.5, 133.6, 133.1, 132.7, 132.5, 130.4, 130.1, 127.2, 124.9, 124.2, 115.2, 105.5, 40.1; HPLC: 98.1 %, Column: Inertsil ODS 3v (150 x 4.6) mm, mobile phase A : 0.01 M KH_2_PO_4_, mobile phase B : acetonirtile, gradient elution T % B: 0/55, 1/55, 8/80, 16/80, 18/55, 20/55, flow rate 1.5 ml/min, UV 210 nm, retention time 6.0 min; HRMS (ESI): calcd for C_15_H_12_ FN2O_4_S (M+H)^+^ 335.0502, found 335.0497.

**5-Fluoro-1-methanesulfonyl-2-(7-methoxy-naphthalen-2-yl)-1*H*-indole (3q):** brown gum; R***_f_*** (20% ethyl acetate/ n-hexane) 0.34; ^1^H NMR (CDCl_3_, 400 MHz) δ 8.12-8.08 (m, 1H), 7.94 (s, 1H), 7.79-7.76 (m, 2H), 7.65-7.63 (m, 1H), 7.24 (s, 1H), 7.20-7.12 (m, 2H), 7.10-7.07 (m,1H), 6.74 (s, 1H), 3.94 (s, 3H), 2.72 (s, 3H); IR (cm^-1^, KBr) 3110, 1358, 1175; m/z (ES Mass) 370.0 (M+1, 100 %); ^13^C NMR (CDCl_3_, 50 MHz) δ 157.7, 134.6, 134.2, 131.7, 129.8, 129.5, 129.3, 127.3, 127.4, 126.2, 124.9, 124.2, 121.0, 120.2,118.8, 112.0, 105.8, 102.0, 56.0, 38.6; HRMS (ESI): calcd for C_20_H_17_FNO_3_S (M+H)^+^ 370.0912, found 370.0899.

**2-(3-Chloro-propyl)-5-fluoro-1-methanesulfonyl-1*H*-indole (3r):** white solid, mp 98-100 °C; R***_f_*** (25 % ethyl acetate/ n-hexane) 0.3; ^1^H NMR (CDCl_3_, 400 MHz) δ 7.96-7.92 (m, 1H), 7.17-7.14 (m, 1H), 7.03-6.99 (m, 1H), 6.49 (s, 1H), 3.60 (t, *J* = 6.7 Hz, 2H), 3.13 ( t, *J* = 6.7 Hz, 2H), 2.99 (s, 3H), 2.26-221 (m, 2H); IR (cm^-1^, KBr) 3421, 1363, 1175; m/z (ES Mass) 290 (M+1, 100 %); ^13^C NMR (CDCl_3_, 50 MHz) δ 155.0, 137.2, 131.2, 129.0, 113.2, 108.4, 107.2, 103.4, 44.6, 38.6, 35.9, 20.5; HRMS (ESI): calcd for C_12_H_14_ ClFNO_2_S (M+H)^+^ 290.0418, found 290.0416.

**1-(5-Fluoro-1-methanesulfonyl-1*H*-indol-2-yl)-cyclohexanol (3s):** low melting solid; R***_f_*** (30% ethyl acetate/ n-hexane) 0.32; ^1^H NMR (CDCl_3_, 400 MHz) δ 8.03-8.01 (m,1H), 7.40-7.37 (m, 1H), 7.16-7.11 ( m, 1H), 6.81 (s, 1H), 4.86 (s, 1H), 3.36 (s, 3H), 2.21-2.18 (m, 2H), 1.89-1.82 (m, 2H), 1.79-1.61 (m, 2H), 1.53-1.50 (m, 2H), 1.26-1.23 (m, 2H); IR (cm^-1^, KBr) 3420, 1356, 1145; m/z (ES Mass) 294 (-OH, 100 %); ^13^C NMR (CDCl_3_, 50 MHz) δ 155.9, 139.2, 131.3, 129.2, 113.3, 108.2, 107.2, 102.2, 68.0, 41.2(C), 38.6, 27.7, 18.3 (2C); HRMS (ESI): calcd for C_15_H_19_FNO_3_S (M+H)^+^ 312.1069, found 312.1064.
